# The Influence of Antisocial Behavior and Callous-Unemotional Traits on Trajectories of School Engagement and Achievement in South-Korean Children

**DOI:** 10.1007/s10964-021-01414-2

**Published:** 2021-03-11

**Authors:** Suhlim Hwang, Rebecca Waller, David J. Hawes, Jennifer L. Allen

**Affiliations:** 1 Department of Psychology and Human Development, UCL Institute of Education, London, UK; 2 Department of Psychology, University of Pennsylvania, Philadelphia, PA USA; 3 School of Psychology, University of Sydney, Sydney, NSW Australia; 4 Department of Psychology, University of Bath, Bath, UK

**Keywords:** Antisocial behavior, Callous-unemotional traits, Verbal ability, Academic performance, School engagement

## Abstract

Poor educational outcomes are common among children with antisocial behavior problems, including among a subgroup of antisocial children with callous-unemotional traits, who show deficits in empathy, guilt, and prosociality. However, few studies have explored the unique contributions of antisocial behavior and callous-unemotional traits to school outcomes and most prior studies have been conducted in Western countries. The current study thus tested associations between callous-unemotional traits, antisocial behavior, and trajectories of school outcomes among South Korean children. Participants aged 10-12 years (*N* = 218; 52% boys) completed questionnaires assessing antisocial behavior, callous-unemotional traits, verbal ability, and school engagement, and teachers provided children’s Math and Korean grades at three time points during a single academic year. Prospective associations were explored in conditional latent growth curve models. Both antisocial behavior and callous-unemotional traits were related to lower school engagement at the start of the academic year, but the magnitude of the associations was greater for callous-unemotional traits, suggesting a greater adverse effect of callous-unemotional traits on engagement than antisocial behavior. Moreover, children with high levels of callous-unemotional traits showed stable and low levels of school engagement. There were no significant predictive associations between antisocial behavior or callous-unemotional traits and trajectories of academic grades. The findings suggest that interventions aimed at improving educational outcomes among antisocial children should be tailored according to the presence of callous-unemotional traits to target the specific needs of individual students, particularly at the start of the school year.

## Introduction

Childhood antisocial behavior is associated with poor educational outcomes that harm children’s adaptive functioning, including poor quality teacher and peer relationships, bullying, truancy, and academic underachievement (McLeod et al., 2012). Antisocial behavior, academic difficulties, and school disengagement constitute major risk factors for later maladjustment, including early school dropout, engagement in criminal offending, and unemployment (Doll et al., 2012). As such, the inter-relationships between these risk factors have long been of interest to researchers, educators, and policy-makers (Wang & Fredricks, 2014). Callous-unemotional traits (i.e., lack of empathy, guilt, restricted interpersonal sensitivity, and a lack of concern for performance) delineate an important subgroup of antisocial children (Waller et al., 2020a) who show an earlier onset and more severe, varied, and persistent forms of antisocial behavior (McMahon et al., 2010). However, very few studies have explored callous-unemotional traits in relation to school engagement or academic outcomes, despite clear potential implications for school-based interventions that could disrupt risky pathways to antisocial behavior (Tyler et al., 2019). This study addresses this gap by investigating the influence of callous-unemotional traits and antisocial behavior on trajectories of academic grades and school engagement in South Korean children over an academic year.

### School Engagement, Antisocial Behavior, and Callous-Unemotional Traits

School engagement and academic achievement are two key competency outcomes that promote developmental pathways towards psychosocial well-being and later employment (Borofsky et al., 2013). School engagement has been conceptualized as a multidimensional construct that includes behavioral (i.e., participation in school-related activities), emotional (i.e., affective responsiveness in school settings) and cognitive (i.e., investment in academic tasks) dimensions (Li & Lerner, 2013), with several attempts to identify associations between antisocial behavior and these distinct dimensions. For example, prior longitudinal studies have found that antisocial behavior predicts less behavioral (Olivier et al., 2020), emotional (Wang & Fredricks, 2014), and cognitive engagement over time (Hirschfield & Gasper, 2011). However, prior studies have not accounted for the influence of callous-unemotional traits. Given that the core features of callous-unemotional traits include motivational deficits in social affiliation (Waller et al., 2020b) and a lack of concern for school performance (Frick et al., 2014a), callous-unemotional traits may be more strongly related to deficient emotional (i.e., caring about relationships with and expectations of others) and cognitive (i.e., investment in academic task) dimensions of school engagement, over and above the presence of antisocial behavior more broadly. Research examining these three types of school engagement in relation to child functioning can provide richer information than focusing on engagement as a unidimensional construct and could help to identify more specific intervention targets for different subtypes of antisocial children (Wang & Eccles, 2013).

To date, the few existing studies examining callous-unemotional traits in relation to school engagement have only focused on the emotional dimension of school engagement, and emerging findings have suggested complex associations between these variables and conduct problems. For example, in a study of children aged 7 to 11 years, school connectedness appeared to have a buffering effect against the development of conduct problems in children with high callous-unemotional traits, with higher levels of school connectedness in the high callous-unemotional traits group than the high callous-unemotional traits and the high conduct problems group (Wall et al., 2016). Another study featuring the same sample identified groups based on levels of callous-unemotional traits only and found that school connectedness acted as a protective factor in the development of callous-unemotional traits, with higher school connectedness in the stable low callous-unemotional traits and decreasing callous-unemotional traits groups (Fanti et al., 2017). Moreover, these studies did not examine the influence of callous-unemotional traits on child school engagement independently of antisocial behavior. Although both callous-unemotional traits and antisocial behavior are related to poor school engagement, the risk pathways may differ for these two constructs, given the concept of equifinality in developmental psychopathology (Frick et al., 2014b). However, no research has been conducted to investigate how these two constructs are differently or interactionally related to school engagement in relation to their pathways, thus it remains unclear if the same intervention target would work for both callous-unemotional traits and antisocial behavior.

### Academic Grades, Antisocial Behavior, and Callous-Unemotional Traits

Another key competency outcome, academic achievement, is traditionally considered to be a defining feature of school success (Furco, 2013). Numerous studies have demonstrated a strong relationship between antisocial behavior and a greater likelihood of academic failure, even when accounting for child gender, socioeconomic status, delinquent peer affiliation, and self-esteem (Jakobsen et al., 2012). Several different explanations have been put forward to explain the link between antisocial behavior and academic failure, but the most robust finding is the presence of verbal deficits in antisocial children (Allen, 2017). Child verbal ability has shown consistent associations with both antisocial behavior and poor academic outcomes (Menting et al., 2011). There also is evidence that interventions aimed at improving academic achievement simultaneously reduced antisocial behavior (Filter & Horner, 2009).

Intriguingly, despite being at high risk for antisocial behavior, children with elevated levels of callous-unemotional traits do not appear to have verbal deficits (Allen et al., 2013). Nonetheless, studies have consistently found a significant association between callous-unemotional traits and poor academic achievement, including when assessed via different statistical methodologies (i.e., variable-centered and person-centered) (Bird et al., 2019; Fanti et al., 2017), different methods of assessing achievement (i.e., teacher ratings, curriculum set assessments, standardized tests) (Ciucci et al., 2014; DeLisi et al., 2011), and different subjects (i.e., Reading, English, Math, Science) (Horan et al., 2016; Vaughn et al., 2011). Current theory highlights the importance of social-motivational factors in the etiology of callous-unemotional traits (Waller & Wagner, 2019). In the school context, children with elevated callous-unemotional traits show lower levels of academic motivation (Allen et al., 2018), a reduced drive for affiliation and approval from parents, teachers (Hwang et al., 2021), and peers (Waller et al., 2020b), and decreased sensitivity to punishment, identified as important factors for promoting engagement and performance in school activities (Hwang et al., 2021). It has therefore been suggested that callous-unemotional traits may be associated with poor school engagement and low grades through different risk pathways than antisocial behavior, given the lack of evidence for a link between callous-unemotional traits and deficits in verbal ability (Allen et al., 2018).

### Research on Antisocial Behavior, Callous-Unemotional Traits and Academic Grades in East-Asian Countries

Most prior studies on callous-unemotional traits, antisocial behavior and academic performance have been cross-sectional and conducted in Western countries, including the UK/Europe and the USA. Thus, it remains uncertain whether findings are generalizable to East Asian countries. In particular, existing studies suggest that there might be no or only a weak association between antisocial behavior and academic achievement among East-Asian youth. For example, one study of Chinese high school students found no significant association between antisocial behavior and academic achievement (Li & Armstrong, 2009). In another study of Chinese elementary school students, child antisocial behavior was not associated with the rate of change for three different academic trajectories (i.e., low stable, high stable, and high/moderate decreasing), but only with a high increasing trajectory, such that less antisocial behavior predicted higher growth rates within this group. Recent studies have also started to examine potential cultural differences in academic motivation and engagement (Qu et al., 2016). East Asian culture, which is based on Confucianism, emphasizes diligence and persistence in pursuing academic achievement as a moral endeavor (Huang & Gove, 2015). This cultural perspective may therefore lead to differences in the development of school engagement and academic achievement. Moreover, one prior study conducted in Singapore showed a non-significant association between callous-unemotional traits and antisocial behavior (Sng et al., 2018), highlighting the need for more studies of students from East Asian cultures to better unpack the relationships between antisocial behavior, callous-unemotional traits, and school outcomes. Given that poor academic functioning is mentioned in the criterion of clinically significant impairment for the diagnosis of conduct disorder in the Diagnostic and Statistical Manual of Mental Disorders (DSM-5; American Psychiatric Association., 2013), examining the association between antisocial behavior and academic failure in a different cultural context may provide useful information for the generalizability of DSM criteria. Furthermore, lack of concern for school performance is included as a core feature of callous-unemotional traits (i.e., the Limited Prosocial Emotions specifier for conduct disorder) in the DSM-5 (American Psychiatric Association., 2013). Therefore, research examining the relationship between specific dimensions of school engagement with antisocial behavior and callous-unemotional traits stands to provide a clearer understanding of the complex interplay between antisocial behavior, callous-unemotional traits, academic motivation and academic competence.

## Current Study

The aim of the current study is to investigate whether antisocial behavior and callous-unemotional traits uniquely predict trajectories of school engagement (i.e., behavioral, emotional, and cognitive engagement) and academic achievement (i.e., Math and Korean) over the course of an academic year. The associations between these constructs were examined in a sample of South Korean students who were in Years 5 and 6 (i.e., aged 10–12 years old), a developmental period when children are likely to show a decrease in academic motivation and achievement, combined with increased antisocial behavior. It was hypothesized that both antisocial behavior and callous-unemotional traits would be uniquely related to lower school engagement and academic grades at the first assessment point. It was further hypothesized that antisocial behavior and callous-unemotional traits would be associated with decreasing trajectories for school engagement and academic grades, but that callous-unemotional traits would be related to steeper decreases in engagement and grades over time than antisocial behavior.

## Methods

### Participants

Participants were 218 children from two different South Korean elementary schools who were 10 to 12 years old (*M* = 11.03, *SD* = .65) at the first assessment (*n* = 113 boys; *n* = 105 girls). All participants were South Korean and recruited from the two highest grades, Years 5 and 6, in the South Korean elementary school system. Twenty-two children (10%) were recipients of free school milk and 17 children (8%) reported that they lived with a single parent. The provision of free school milk is an index of low socioeconomic status in South Korea (South Korea Welfare Service 2019). All elementary school students in South Korea receive free school meals regardless of family income.

### Procedure

Following university ethics board approval, written consent was obtained from parents. Of the 274 parents informed of the study aim and processes, 56 declined to give consent for their child’s participation (20%). The 218 students with parental consent were then approached to obtain their written informed assent. After a baseline assessment (Time 1), children completed two follow-up assessments (Time 2 and Time 3) at 4.5-month intervals. South Korean schools operate a two-term school year that runs from March to February of the following year. An academic assessment is conducted at the start of the new academic year (i.e., March) and two further formal academic assessments are conducted at the end of each semester (i.e., July and December, respectively). Data collection for the current study followed this same schedule. Students completed questionnaires during regular classes at all three assessment points. Retention rates were high (98.6% at Time 2 and 97.7% at Time 3); only seven children missed one or both follow-ups, leading to final sample of 211 children included in the analysis (96.8%; 108 boys, 103 girls).

### Measures

#### Callous-unemotional traits and antisocial behavior

At Time 1, children completed the revised University of New South Wales (UNSW) callous-unemotional traits index (Hwang et al., 2020) which features combined items from the Strengths and Difficulties Questionnaire (SDQ; Goodman, 1997) and the Antisocial Process Screening Device (APSD; Frick & Hare, 2001). The 9-item measure assesses child lack of guilt and callousness (e.g., “I feel bad or guilty when I do something wrong”, “I try to be nice to others and care about their feelings” [reverse scored]) using a 3-point scale (0 = not true; 1 = somewhat true; 2 = certainly true). The revised scale showed high internal consistency (*α* = .73) and evidence for construct validity, including significant associations with antisocial behavior and insensitivity to teacher discipline (Hwang et al., 2020).

#### Antisocial behavior

At Time 1, child antisocial behavior was assessed using self-report on the revised UNSW antisocial behavior index (Hwang et al., 2020). Three items from the SDQ and six items from the APSD that assess aggression and externalizing symptoms were combined (e.g., ‘I fight a lot’, ‘I am often accused of lying or cheating’) and scored on a 3-point scale (0 = not true; 1 = somewhat true; 2 = certainly true). The revised scale showed good internal consistency (*α* = .72) and its construct validity was evidenced in a previous study that found significant associations with callous-unemotional traits and more frequent use of teacher harsh discipline (Hwang et al., 2020).

#### School engagement

The 19-item School Engagement Scale (SES; Fredricks et al., 2005) was used to assess children’s perceptions of their school engagement, including levels of teacher support, peer support, and academic challenge. Items are rated on a 5-point Likert scales from 1 (never) to 5 (all of the time). The 19 items form three factors: behavioral engagement (5 items), emotional engagement (6 items), and cognitive engagement (8 items). Prior research supports the construct validity of the individual factors, with higher cognitive engagement linked to academic task challenge and more teacher support linked to emotional engagement in 8–11 year olds (Fredricks et al., 2005). In the current study, separate factor scores showed high internal consistency at each time point: behavioral engagement (Time 1, *α* = .78; Time 2, *α* = .79; Time 3, *α* = .78), emotional engagement (Time 1, *α* = .93; Time 2, *α* = .91; Time 3, *α* = .93), and cognitive engagement (Time 1, *α* = .86; Time 2, *α* = .87; Time 3, *α* = .87). The model fit of the three-factor model in the current sample was also adequate (CFI = .93, TLI = .92, RMSEA = .07, SRMR = .05).

#### Academic performance

Math and Korean attainment scores based on tests derived and set by the Korean government and teachers were used to measure child academic achievement. There are three exams in Korean primary schools during the academic year. The first exam is set by the Korean government to assess the scholastic level of students and identify whether students are ready to begin the new school year in March (i.e., Time 1). The next two exams are set separately by schools to assess students’ achievement at the end of each term and are conducted in July (Time 2) and December (Time 3). Consistent with prior studies, Math and Korean exam scores from each time point were converted to standardized z-scores to allow for comparison (Hsiung, 2010).

#### Verbal ability

At Time 1, child verbal ability was assessed using a verbal fluency test (Shao et al., 2014). The test consists of two tasks: category fluency and letter fluency. The category fluency task asks children to produce as many words as possible in one minute for two categories (animals and food). The letter fluency task is conducted in the same way but, this time, starting with a specific letter (Korean letters, ㄱ and ㅅ). Children’s scores were computed as the number of unique correct words in the four tasks excluding the names of people or places. Prior research has established the validity of this task in relation to child outcomes, with poor verbal ability scores related to attention deficit/hyperactivity disorder (Hurks et al., 2004) and prenatal alcohol exposure (Rasmussen & Bisanz, 2009). The combined scale of category fluency and letter fluency tasks showed good internal consistency in the current sample (*α* = .71).

#### Sociodemographic information

Children reported their age and gender at Time 1 and teachers provided information on single parent status and eligibility for free school milk.

### Data Analysis

First, a latent growth curve model (LGCM) was fitted to estimate baseline levels of the three dimensions of school engagement (i.e., behavioral, emotional, and cognitive) and academic grades (i.e., Math and Korean) (i.e., intercept) and whether linear change (i.e., slope) accounted for observed changes in these constructs across time. Due to the small number of measurement occasions (i.e., three), a quadratic term was not used to avoid overfitting. Slope factor loadings were fixed to 0 for Time 1 and to 1 and 2 for Time 2 and Time 3 to reflect differences in assessment ages. Second, to test whether callous-unemotional traits and antisocial behavior at Time 1 were related to change in school engagement or academic grades across time, the latent growth parameters were regressed onto scores for callous-unemotional traits and antisocial behavior, with separate models for school engagement and academic grades. A product term between the two (i.e., callous-unemotional traits × antisocial behavior) was also included to test for potential interaction effects. Child verbal ability, age, gender, family type, and free school milk were included as covariates in each model with regressions on all latent growth parameters in both models. As the sample was nested across 11 classrooms, dummy variables for each classroom were included to control for potential classroom effects. Model fit was assessed by the following fit indices: Chi-Square Test of Model Fit, CFI, TLI, SRMR, and RMSEA. CFI and TLI values higher than .90 and SRMR and RMSEA values lower than .10 indicate acceptable fit (Hu & Bentler, 1999). All models were fitted using R software (R Core Team 2013).

## Results

### Descriptive Statistics

Descriptive statistics for all study variables are presented in Table 1 and bivariate correlations are presented in Table 2. All dimensions of school engagement were moderately-to-highly stable across all three time points (range, *r*s = .39 − .74, *p*s < .01). Consistent with expectations, callous-unemotional traits were related to higher levels of antisocial behavior at Time 1. Higher callous-unemotional traits were moderately-to-strongly correlated with lower school engagement across all three dimensions (range, *r*s = −.52 − −.28, *p*s < .01). Higher levels of antisocial behavior were also moderately correlated with lower behavioral engagement across time (range, *r*s = −.41 − −.36, *p*s < .01), but only modestly correlated with emotional and cognitive engagement (range, *r*s = −.32 − −.24, *p*s < .01). The results of Fisher’s *r* to z transformations showed that the correlations of callous-unemotional traits with emotional engagement at Time 1 and cognitive engagement at Time 1 and Time 2 were significantly larger in magnitude than those for antisocial behavior (range, *z*s = −2.88 − −2, *p*s < .05). Higher verbal ability at Time 1 was related to higher emotional engagement at Time 1 and Time 2, but not to behavioral and cognitive engagement at either Time 2 or Time 3 (Table 2).

**Table 1:** Descriptive statistics for the sample across the time points

	Time 1					Time 2				Time 3			
Variable	*N*	*M*	*SD*	range		*N*	*M*	*SD*	range	*N*	*M*	*SD*	range
Callous-unemotional traits	214	6.68	3.01	0 – 18		–	–	–	–	–	–	–	–
Antisocial behavior	213	2.59	2.47	0 – 17		–	–	–	–	–	–	–	–
Verbal ability	218	40.77	11.74	13 – 70		–	–	–	–	–	–	–	–
Behavioral engagement	214	20.22	2.94	9 – 25		215	19.74	3.24	10 – 25	212	19.51	3.21	9 – 25
Emotional engagement	216	21.94	5.43	6 – 30		215	22.25	5.30	6 – 30	211	21.80	5.52	6 – 30
Cognitive engagement	215	24.37	6.54	8 – 40		215	24.33	6.49	8 – 40	211	23.29	6.70	8 – 40
Math grade	218	0	1	−2.75 – 1.80		218	0	1	−3.07 – 1.53	218	0	1	−3.12 – 1.30
Korean grade	218	0	1	−3.67 – 1.41		218	0	1	−3.53 – 1.65	218	0	1	−3.40 – 1.30

**Table 2:** Bivariate cross-sectional and longitudinal correlations among study variables

Variable	1	2	3	4	5	6	7	8	9	10	11	12	13	14	15	16	17
Callous-unemotional traits																	
Antisocial behavior	0.20**																
Verbal ability	−10	0.03															
Behavioral Engagement Time 1	−50**	−41**	0.09														
Behavioral Engagement Time 2	−37**	−40**	0.11	0.69**													
Behavioral Engagement Time 3	−30**	−36**	0.13	0.61**	0.72**												
Emotional Engagement Time 1	−42**	−28**	0.08	0.50**	0.42**	0.39**											
Emotional Engagement Time 2	−44**	−27**	0.14*	0.48**	0.61**	0.51**	0.74**										
Emotional Engagement Time 3	−33**	−24**	0.14*	0.41**	0.47**	0.56**	0.65**	0.69**									
Cognitive Engagement Time 1	−52**	−32**	0.07	0.54**	0.46**	0.35**	0.57**	0.54**	0.46**								
Cognitive Engagement Time 2	−49**	−25**	0.11	0.45**	0.59**	0.45**	0.47**	0.58**	0.50**	0.71**							
Cognitive Engagement Time 3	−28**	−27**	0.07	0.34**	0.46**	0.43**	0.39**	0.46**	0.54**	0.55**	0.72**						
Math grade Time 1	−16*	−03	0.12	0.15*	0.15*	0.18**	0.05	0.04	0.10	0.04	0.18**	0.19**					
Math grade Time 2	−03	−11	0.06	0.16*	0.25**	0.25**	0.00	0.12	0.18**	0.08	0.17*	0.23**	0.45**				
Math grade Time 3	−06	−13	0.05	0.18**	0.09	0.02	0.11	0.06	−01	0.14*	0.08	0.01	0.26**	0.21**			
Korean grade Time 1	−13	−10	0.23**	0.14*	0.10	0.17*	0.02	0.08	0.12	0.07	0.17*	0.14*	0.44**	0.39**	0.14*		
Korean grade Time 2	−10	−14*	0.21**	0.16*	0.30**	0.28**	0.07	0.15*	0.16*	0.14*	0.28**	0.26**	0.33**	0.50**	0.24**	0.45**	
Korean grade Time 3	−06	−12	0.02	0.20**	0.09	0.04	0.11	0.08	0.04	0.17*	0.12	0.03	0.17*	0.07	0.77**	0.10	0.21**

Differences between correlations for callous-unemotional traits and antisocial behavior were examined using the Fisher r-to-z transformations and significantly different correlations are presented in italics

*p < 0.05. **p < 0.01

In terms of academic achievement, Korean and Math scores were modestly-to-moderately stable across time points (range, *r*s = .21 − .45, *p*s < .01). There were inconsistent associations for callous-unemotional traits and antisocial behavior: callous-unemotional traits were significantly related to lower Math grades at Time 1 and antisocial behavior was significantly related to lower Korean grades at Time 2. Consistent with expectations, verbal ability was related to higher Korean grades, but was unrelated to Math grades. Greater behavioral and cognitive engagement were also significantly related to higher Math and Korean grades both cross-sectionally and longitudinally (range, *r*s = .14 − .30, *p*s < .05). Emotional engagement at Time 3 was only correlated with Math and Korean grades at Time 2, and emotional engagement at Time 2 was only cross-sectionally correlated with Korean grades at Time 2 (Table 2).

### Unconditional Latent Growth Curve Models

The LGCM for the school engagement variables showed acceptable fit to the data (χ^2^ (*df* = 18) = 76.47, *p* < .001; CFI = .95; TLI = .91, SRMR = .04; RMSEA = .13). Behavioral (*B* = −.37, *SE* = .10, *β* = −.39, *p* < .001) and cognitive engagement (*B* = −.55, *SE* = .21, *β* = −.25, *p* < .001) decreased significantly over time, but there was no significant reduction in emotional engagement (*B* = −.09, *SE* = .16, *β* = −.11, *p* = .547). The intercepts for all three engagement dimensions were positively correlated with each other (range, *r*s = .64 − .74, *p*s < .001). Likewise, the slopes of all three dimensions of school engagement were significantly positively related to each other (range, *r*s = .38 − .75, *p*s < .001). The LCGM for the academic variables also showed good fit to the data (χ^2^ (*df* = 7) = 12.48, *p* = .086; CFI = .99.; TLI = .98, SRMR = .03; RMSEA = .06). However, neither Math grades (*B* = .01, *SE* = .03, *β* = .01, *p* = .92) nor Korean grades (*B* = .01, *SE* = .04, *β* = .02, *p* = .90) showed significant change over time. The intercept and slope for both grades were not correlated with one another, such that starting levels were not related to change over time. However, there were significant cross-subject correlations, such that the starting points (i.e., intercepts) of Math and Korean grades were highly related to each other (*r* = .76, *p* < .001). Likewise, the slopes for Math and Korean grades were significantly positively related to each other (*r* = .91, *p* < .001).

### Callous-Unemotional Traits and Antisocial Behavior as Predictors of the Latent Growth Curve Models

**Fig. 1:**
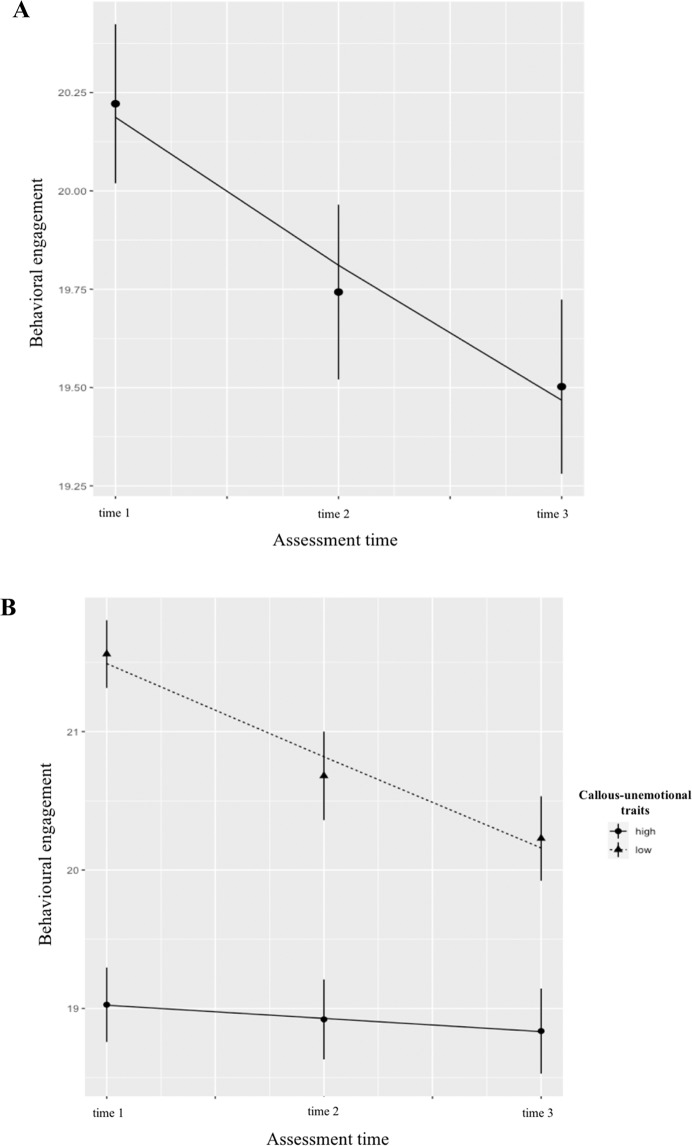
Main effect of callous-unemotional traits predicting the intercept and slope of the behavioral engagement trajectory. **A** *Note*. Change in behavioral engagement in the total sample. There was a significant decrease in behavioral engagement over time (intercept, *B* = 20.22, *SE* = .21, *β* = 7.57*, p* < .001; slope, *B* = −.37, SE = .10, *β* = −.39*, p* < .001). **B** *Note*. Change in behavioral engagement in sample split according to level of callous-unemotional traits (high vs. low based on median split). Children with high callous-unemotional traits showed stable low behavioral engagement (intercept, *B* = 19.15, *SE* = .32, *β* = −.28, *p* < .001; slope, *B* = −.11, *SE* = .13, *β* = −.03, *p* = .38), whereas children with low levels of callous-unemotional traits showed significantly higher baseline levels of behavioral engagement that decreased significantly over time (intercept, *B* = 22.14, *SE* = .03, *β* = .32, *p* < .001; slope, *B* = −.55, *SE* = .18, *β* = −.14, *p* < .001)

**Fig. 2:**
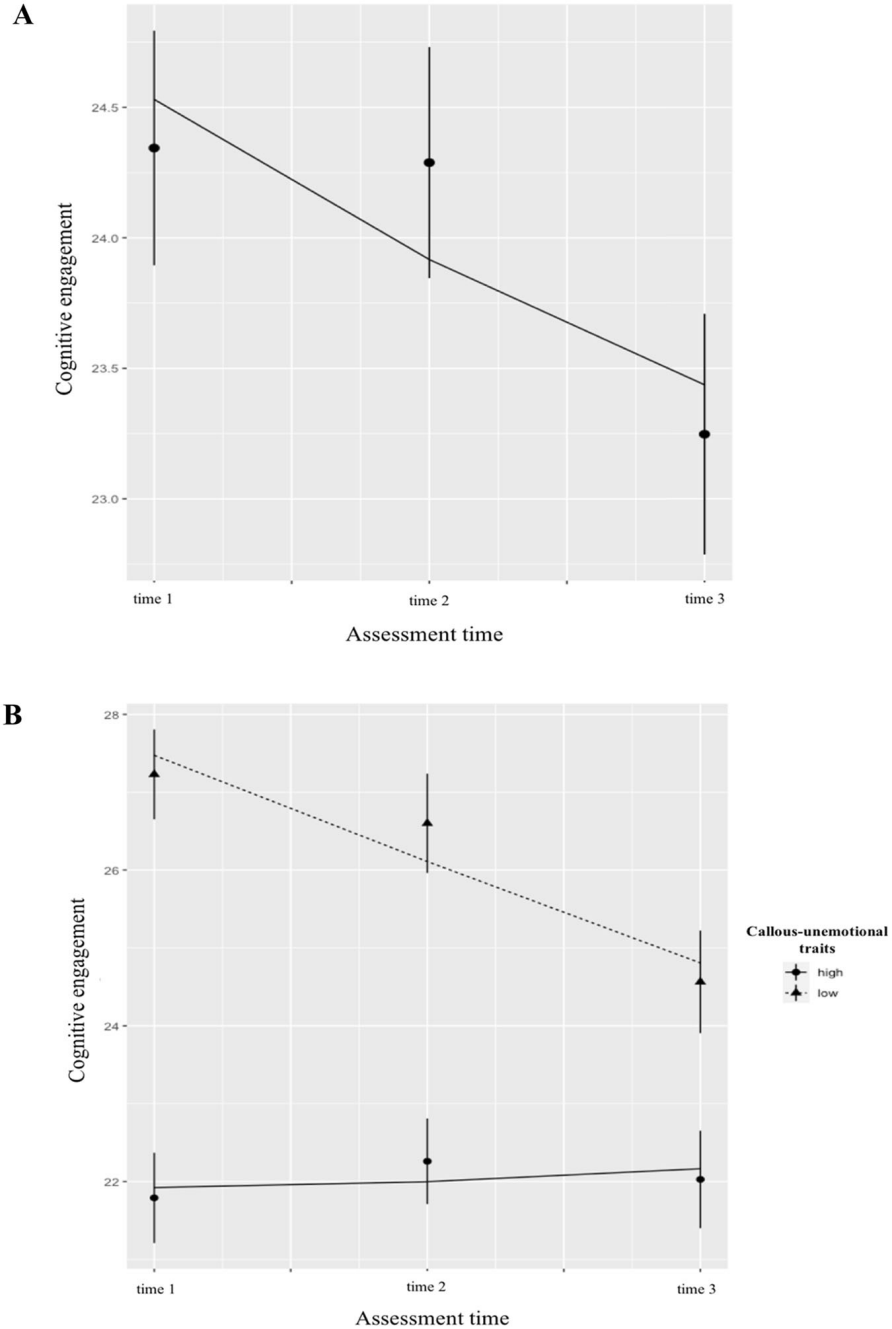
Main effect of callous-unemotional traits predicting the intercept and slope of the cognitive engagement trajectory. **A** *Note*. Change in cognitive engagement in the total sample. There was a significant decrease in cognitive engagement over time (intercept, *B* = 24.58, *SE* = .45, *β* = 4.21*, p* < .001; slope, *B* = −.55, *SE* = .21, *β* = −.25*, p* < .01). **B** *Note*. Change in cognitive engagement in sample split according to level of callous-unemotional traits (high vs. low based on median split). Children with high CU traits showed stable low cognitive engagement (intercept, *B* = 21.83, *SE* = .72, *β* = −.30, *p* < .001; slope, *B* = .08, *SE* = .29, *β* = .01, *p* = .78), whereas children with low levels of callous-unemotional traits showed significantly higher baseline levels of cognitive engagement that decreased significantly over time (intercept, *B* = 28.74, *SE* = 1.04, *β* = .33, *p* < .001; slope, *B* = −1.31, *SE* = .42, *β* = −.16, *p* < .001)

First, callous-unemotional traits and antisocial behavior were both related to lower starting levels for all three engagement dimensions (range, *β*s = −.52 − −.14, *p*s < .05), although the magnitude of the effects for callous-unemotional traits was larger in each case (Table 3). In addition, callous-unemotional traits, but not antisocial behavior, predicted slope factors for behavioral and cognitive school engagement (Table 3). These relationships were probed by plotting the behavioral engagement and cognitive engagement trajectories for children with high versus low levels of callous-unemotional traits, using a median split. Children with low callous-unemotional traits had higher initial levels of behavioral engagement (*B* = 22.14, *SE* = .03, *β* = .32, *p* < .001) that decreased significantly over the academic year (*B* = −.55, *SE* = .18, *β* = −.14, *p* < .001; Fig. 1). In contrast, children with high levels of callous-unemotional traits had low initial levels of behavioral engagement (*B* = 19.15, *SE* = .32, *β* = −.28, *p* < .001) and showed stable low levels of behavioral engagement across time (*B* = −.11, *SE* = .13, *β* = −.03, *p* = .38; Fig. 1). Children with low callous-unemotional traits had also showed significantly higher initial levels of cognitive engagement (*B* = 28.74, *SE* = 1.04, *β* = .33, *p* < .001) that decreased significantly over the academic year (*B* = −1.31, *SE* = .42, *β* = −.16, *p* < .001; Fig. 2). In contrast, children with high levels of callous-unemotional traits had low initial levels of cognitive engagement (*B* = 21.83, *SE* = .72, *β* = −.30, *p* < .001) and showed stable low levels of cognitive engagement across time (*B* = .08, *SE* = .29, *β* = .01, *p* = .775; Fig. 2). However, children with high callous-unemotional traits showed significantly lower levels of behavioral and cognitive engagement than children with low callous-unemotional traits across all time points, despite the decreasing engagement in children with low callous-unemotional traits over the three time points.

**Table 3:** Callous-unemotional traits and antisocial behavior predicting school engagement growth trajectories

Model 1 (school engagement)																		
	Behavioral engagement						Emotional engagement						Cognitive engagement					
	Intercept			Slope			Intercept			Slope			Intercept			Slope		
Predictor	B (SE)	*β*	*p*	B (SE)	*β*	*p*	B (SE)	*β*	*p*	B (SE)	*β*	*p*	B (SE)	*β*	*p*	B (SE)	*β*	*p*
Callous-unemotional traits	−0.44 (0.06)	−0.47	<0.001	0.09 (0.03)	0.28	0.008	−0.65 (0.12)	−0.41	<0.001	0.04 (0.06)	0.19	0.479	−1.09 (0.14)	−0.52	<0.001	0.23 (0.08)	0.32	0.001
Antisocial behavior	−0.37 (0.07)	−0.35	<0.001	0.02 (0.04)	0.05	0.661	−0.38 (0.14)	−0.21	0.007	0.04 (0.07)	0.16	0.575	−0.34 (0.17)	−0.14	0.041	−0.03 (0.10)	−0.03	0.746
Verbal ability	0.01 (0.02)	0.06	0.340	0.03 (0.01)	0.35	0.002	0.06 (0.03)	0.16	0.040	0.02 (0.02)	0.46	0.115	0.04 (0.04)	0.08	0.270	0.02 (0.02)	0.11	0.280
Age	0.23 (0.33)	0.06	0.487	−0.41 (0.19)	−0.30	0.030	−1.35 (0.67)	−0.19	0.043	−0.02 (0.33)	−0.02	0.956	−1.48 (0.79)	−0.16	0.060	−0.21 (0.45)	−0.06	0.645
Gender	0.24 (0.34)	0.04	0.491	0.02 (0.19)	0.01	0.907	0.93 (0.68)	0.10	0.173	0.12 (0.33)	0.10	0.723	1.13 (0.88)	0.10	0.162	−0.29 (0.46)	−0.06	0.524
Family type	−0.38 (0.68)	−0.04	0.574	0.12 (0.38)	0.04	0.760	−1.75 (1.35)	−0.10	0.193	0.11 (0.66)	0.05	0.870	−2.03 (1.59)	−0.09	0.203	0.08 (0.91)	0.01	0.933
Free school milk	−0.40 (0.64)	−0.04	0.528	−0.01 (0.36)	−0.00	0.971	0.83 (1.27)	0.05	0.516	1.06 (0.62)	0.53	0.088	1.37 (1.50)	0.07	0.362	0.37 (0.86)	0.05	0.664

Dummy codes for classroom effects were all entered as covariates, but are not shown in the table. A two-way interaction between callous-unemotional traits and antisocial behavior was not significantly related to any of the slopes, thus not shown in the table for parsimony

**Fig. 3:**
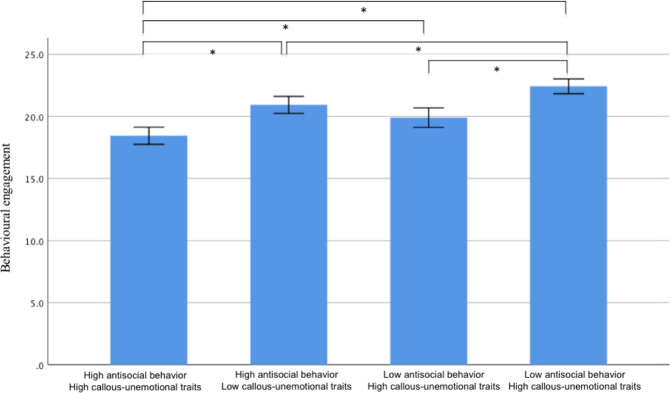
Differential associations between antisocial behavior and the intercepts of the behavioral engagement trajectory for children with high and low levels of callous-unemotional traits. *Note*. Each intercept for the behavioral engagement trajectory is presented according to groups based on the levels of antisocial behavior and callous-unemotional traits. *p < .05 (Bonferroni corrected)

A two-way interaction between callous-unemotional traits and antisocial behavior was also examined in relation to the slope and intercept factors for the engagement dimensions. The only significant interaction was in the prediction of the intercept for behavioral engagement (*B* = .40, *SE* = .19, *β* = .15, *p* = .04). This interaction was probed by comparing the intercept of behavioral engagement in children with high versus low antisocial behavior and high versus low callous-unemotional traits (i.e., four groups; see Fig. 3). Children with high antisocial behavior and high callous-unemotional traits showed the lowest level of behavioral engagement at baseline, with lower behavioral engagement than all other groups (range *t* = −7.96 − −2.97, *p*s < .05). In contrast, children with low antisocial behavior and low callous-unemotional traits showed the highest starting levels of behavioral engagement (range *t* = −7.96 − −2.88, *p*s < .05) (see Table S1 for all group comparisons).

There were no significant relationships between callous-unemotional traits and antisocial behavior and either the intercept or slope factors for the Math or Korean grades, nor any interaction of the two in relation to the LGCM. However, higher verbal ability was significantly related to higher Math and Korean grades at baseline, whereas receiving free school milk was related to lower Math and Korean grades at baseline (Table 4).

**Table 4:** Callous-unemotional traits and antisocial behavior predicting academic grades growth trajectories

Model 2 (academic grades)												
	Math grades						Korean grades					
	Intercept			Slope			Intercept			Slope		
Predictor	B (SE)	*β*	*p*	B (SE)	*β*	*p*	B (SE)	*β*	*p*	B (SE)	*β*	*p*
Callous-unemotional traits	−0.05 (0.02)	−0.17	0.055	−0.01 (0.01)	−0.07	0.523	−0.03 (0.02)	−0.13	0.164	−0.02 (0.01)	−0.18	0.190
Antisocial behavior	−0.02 (0.03)	−0.05	0.577	−0.02 (0.01)	−0.14	0.222	−0.01 (0.03)	−0.05	0.649	−0.01 (0.02)	−0.09	0.521
Verbal ability	0.01 (0.01)	0.19	0.038	0.01 (0.00)	0.21	0.076	0.02 (0.01)	0.35	<0.001	0.00 (0.00)	0.00	0.982
Age	0.08 (0.14)	0.07	0.560	0.08 (0.06)	0.17	0.214	0.01 (0.14)	0.00	0.970	0.07 (0.08)	0.15	0.376
Gender	−0.21 (0.14)	−0.13	0.142	0.04 (0.07)	0.07	0.518	0.07 (0.14)	0.05	0.619	0.10 (.08)	0.18	0.203
Family type	0.21 (0.28)	0.07	0.458	0.15 (0.13)	0.13	0.251	0.14 (0.28)	0.05	0.627	0.16 (.16)	0.14	0.322
Free school milk	−0.58 (0.27)	−0.21	0.031	−0.12 (0.13)	−0.12	0.342	−0.64 (0.27)	−0.25	0.018	0.21 (.15)	0.20	0.172

Dummy codes for classroom effects were all entered as covariates but are not shown in the table. A two-way interaction between callous-unemotional traits and antisocial behavior was not significantly related to any of the slopes, thus it is not shown in the table for parsimony

## Discussion

Previous studies have found a strong association between antisocial behavior and poor engagement and performance in school. Current theory predicts that risk pathways for poor school outcomes may differ for callous-unemotional traits and antisocial behavior. There is, however, a lack of research investigating how these two constructs are differently or interactionally related to school engagement in relation to these risk pathways. Furthermore, past research has focused solely on emotional engagement even though callous-unemotional traits may be more strongly related to the emotional and cognitive dimensions of engagement, over and above the presence of antisocial behavior and verbal ability. The findings showed that both antisocial behavior and callous-unemotional traits are uniquely related to trajectories of children’s school engagement and academic grades. Furthermore, the results supported the hypothesis that antisocial behavior and callous-unemotional traits are uniquely related to distinct dimensions of school engagement.

In the current study, both antisocial behavior and callous-unemotional traits were significantly related to lower levels of school engagement across all three dimensions at the start of the academic year. However, the magnitude of the associations was greater for callous-unemotional traits, especially in relation to emotional and cognitive engagement, such that callous-unemotional traits were more strongly associated with lower emotional and cognitive engagement than antisocial behavior. Given that the core features of callous-unemotional traits include interpersonal callousness, a reduced drive for affiliation, and decreased sensitivity to others’ emotions (Waller et al., 2020b), it stands to reason that callous-unemotional traits may be related to less emotional engagement, which encapsulates an overall sense of caring about the wishes and expectations of teachers and peers (Hirschfield & Gasper, 2011). The significant association between callous-unemotional traits and lower cognitive engagement is also consistent with the view that children with elevated callous-unemotional traits show poor academic outcomes due to a lack of motivation to invest in academic tasks as opposed to deficits in cognitive ability (Allen et al., 2018).

In terms of the changes in behavioral and cognitive school engagement over the course of the academic year, antisocial behavior was not significant predictor, with the same decreasing levels of engagement among children regardless of the levels of antisocial behavior. This finding, overall, is consistent with prior literature suggesting that child school engagement tends to show continuous declines across the school year (Wang & Eccles, 2012). As the school year progresses, children may have negative experiences (i.e., decreased teacher support, increased academic pressure, peer problems) that dampen their initial enthusiasm and involvement in school activities (Smith et al., 2010). Thus, researchers and policy makers have promoted school-based interventions aimed at enhancing teacher support, peer relationships, and academic ability to increase children’s school engagement (Fredricks et al., 2019). The results also pointed to steadily decreasing trajectories of behavioral and cognitive engagement. However, in contrast to the hypothesis in the current study and prevailing views of antisocial behavior as a significant factor impeding school engagement (Wang & Fredricks, 2014), decreasing trajectories of school engagement in our study were not aggravated by levels of antisocial behavior. This is consistent with findings from a previous study of secondary school children, in which child aggression was related to lower school engagement at the initial assessment, but not to changes in engagement over 4 years (Engels et al., 2017). It seems that although children with antisocial behavior have lower initial levels of engagement, the magnitude of consequent decreases in engagement are similar to those of children with lower levels of antisocial behavior. This suggests that a universal preventive intervention approach to promoting school engagement is warranted regardless of levels of antisocial behavior. Future research should examine the association between antisocial behavior and school engagement at an earlier stage of schooling, to help identify potential targets of intervention to prevent lower levels of initial engagement for antisocial children. It also highlights challenges for teachers in maintaining the engagement of all their students over time following the initial rush of excitement that accompanies the commencement of each school year.

In contrast to the results for antisocial behavior, findings indicated that callous-unemotional traits were related to different trajectories for school engagement. Children low in callous-unemotional traits showed higher initial levels of behavioral and cognitive engagement that decreased over time, whereas children with high callous-unemotional traits showed stable low levels of engagement for both dimensions. These findings suggest that children with high callous-unemotional traits may be less susceptible to contextual factors that contribute to a decline in school engagement, such as the cumulative effects of experience of failure, increasing pressure to achieve, or decreased teacher support (Smith et al., 2010). However, children with high callous-unemotional traits showed significantly lower initial levels of school engagement, and their overall engagement levels were lower than those of children with low callous-unemotional traits across all time points. Thus, the lower school engagement of children high in callous-unemotional traits may be driven by different factors than for children low in callous-unemotional traits, and likely reflects a reduced drive for achievement (Frick et al., 2014a, b) and social approval (Waller et al., 2020b). These findings also suggest that typical interventions aiming to promote child school engagement, such as those targeting child academic ability, may not be effective for children with high callous-unemotional traits. There is some initial evidence suggesting that promoting teacher-child relationship quality (Allen et al., 2018) and the effective use of discipline and reward-based classroom management strategies (Hwang et al., 2021) may facilitate academic engagement and performance in children with high callous-unemotional traits. However, mechanisms of change in response to intervention targeting school performance and engagement for children with different levels of callous-unemotional traits has yet to be formally tested within a randomized controlled trial design.

In bivariate correlations, callous-unemotional traits showed a significant negative correlation with Math attainment at the start of the academic year, while antisocial behavior showed a significant negative association with Korean attainment at the start of the academic year. However, in contrast to the hypothesis in the current study and prior literature showing significant associations of poor academic achievement with both antisocial behavior (McLeod et al., 2012) and callous-unemotional traits (Bird et al., 2019), there were no significant associations between antisocial behavior, callous-unemotional traits and Math or Korean grades. In contrast to prior studies, child verbal ability was accounted for given its status as a well-established risk factor for poor school outcomes in antisocial children (Allen, 2017) and therefore the possibility that verbal deficits may explain the non-significant effects for antisocial behavior. Moreover, the current study was conducted over one academic year, whereas prior longitudinal studies that found a significant link between antisocial behavior and academic failure had follow-ups of 4–5 years (Stipek & Miles, 2008; Zhou et al., 2010). Finally, prior studies that considered both callous-unemotional traits and antisocial behavior in relation to academic grades were either cross-sectional (e.g., Bird et al., 2019; Ciucci et al., 2014), or failed to control for baseline levels of achievement (e.g., Fanti et al., 2017; Horan et al., 2016).

The findings may also be explained by cultural differences, as prior studies that have explored the link between antisocial behavior and academic performance have predominantly been conducted in Western countries. In support of this assertion, prior studies of Chinese children either found no association (Li & Armstrong, 2009) or a weak association between antisocial behavior and academic achievement, such that neither aggression nor externalizing problems were related to the rate of change for any of the different trajectories of academic achievement (i.e., high/moderate decreasing, high stable, and low stable), but only to the high increasing trajectory (i.e., less growth) over a 5 year period (Fu et al., 2016). These findings may be attributed to Chinese cultural values, which emphasize a strong work ethic and high achievement levels of academic engagement, and performance, which may leave students without the time or motivation to engage in antisocial behavior. Similarly, South Korea has a highly competitive culture regarding education, with a strong sense of obligation to accomplish a high level of scholastic achievement (Song et al., 2015).

The current findings should be considered in the context of several limitations. First, child antisocial behavior, callous-unemotional traits, and school engagement were assessed solely based on child report, thus the reported associations may be inflated by shared method variance. Future research should strive to include teacher and parent perspectives. Second, despite the consistent association between antisocial behavior and poor verbal ability in prior Western literature (Allen, 2017), there was no significant relationship between antisocial behavior and verbal ability. Future research would benefit from the inclusion of a more formal, standardized measure of verbal ability, such as the verbal scales of the Wechsler Intelligence Scale for Children (WISC-V; Wechsler, 2014) which has been validated in South Korean children (Kim & Choi, 2014). This would also help to clarify whether the current findings reflect measurement or cultural differences (see Ogata, 2014). However, the verbal ability measure used in the present study has demonstrated its validity in prior research (Hurks et al., 2004; Rasmussen & Bisanz, 2009). It has previously shown good internal consistency, and verbal ability scores did predict academic grades in the current study, providing evidence for its construct validity.

Third, there may be differences between children who participated versus those who did not (20%), and the distribution for antisocial behavior was positively skewed. Thus, these factors should be taken into account when considering the generalizability of findings. Recent evidence in support of cultural influences suggests that the manifestation (Allen et al., 2021) and correlates (Hwang et al., 2020) of callous-unemotional traits in children from East Asian countries may differ from those in Western countries (see Sng et al., 2020). In addition, the educational system of Korea is based on Confucianism, which encourages students to feel a strong sense of obligation to pursue scholastic achievement. This influence may have impacted the findings for academic achievement relative to the findings of studies conducted in Western cultures (Song et al., 2015). Finally, there may be greater malleability in academic performance across longer time spans within or between different developmental stages (e.g., early vs. middle childhood), rather than within a single school year (Kasanen et al., 2009). Thus, future research should aim to investigate the relationships between callous-unemotional traits, antisocial behavior and school engagement and performance over longer periods of time and across different stages of schooling, particularly during earlier stages of schooling where greater change in performance may occur (Kasanen et al., 2009).

## Conclusion

Prior work had yet to fully identify the unique role of CU traits on trajectories of poor school outcomes when accounting for verbal ability and antisocial behavior. The current study addressed these limitations by examining the unique association between callous-unemotional traits and antisocial behavior in predicting school engagement and academic grades trajectories during early adolescence. As hypothesized, antisocial behavior and callous-unemotional traits were detrimental to school outcomes even after accounting for their overlap, verbal ability, and indices of social disadvantage. Moreover, findings showed the importance of investigating inter-individual differences relating to changes in school engagement over time, with findings suggesting that careful design and implementation of intervention strategies aimed at promoting school engagement and performance is needed, based on an individualized needs assessment accounting for children’s unique temperamental characteristics. The findings also provided further evidence for potential East-West cultural differences in relation to the correlates of callous-unemotional traits and antisocial behavior, with no significant associations present between antisocial behavior, callous-unemotional traits, and academic grades in South Korean children. The current findings advance our knowledge of the pathways between antisocial behavior, callous-unemotional traits, school disengagement and academic failure in early adolescence, which together have important long-term implications for later life, including unemployment, criminal offending, and well-being.

## Supplementary information

Table S1
