# Supplementary material for: The Influence of Antisocial Behavior and Callous-Unemotional Traits on Trajectories of School Engagement and Achievement in South-Korean Children
Source: J Youth Adolesc. 2021 Mar 11;50(4):788–802. doi: 10.1007/s10964-021-01414-2 (PMC7979584; doi:10.1007/s10964-021-01414-2)
Supplement: Supplementary file 1 — Table S1 [file 10964_2021_1414_MOESM1_ESM.docx]

Table S1

*ANOVA results comparing identified groups on intercepts for behavioral engagement*

| Comparison | | Intercept difference | SE | df | t | *p* |
| --- | --- | --- | --- | --- | --- | --- |
| HighAB HighCU | HighAB LowCU | -2.50 | .46 | 210 | -5.44 | <.001 |
|  | LowAB HighCU | -1.46 | .49 | 210 | -2.97 | .020 |
|  | LowAB LowCU | -3.99 | .50 | 210 | -7.96 | <.001 |
| HighAB LowCU | LowAB HighCU | 1.04 | .51 | 210 | 2.05 | .247 |
|  | LowAB LowCU | -1.49 | .52 | 210 | -2.88 | .026 |
| LowAB High CU | LowAB LowCU | -2.52 | .54 | 210 | -4.65 | <.001 |

*Note.* AB = Antisocial behavior, CU = Callous-unemotional traits. Post-hoc pairwise comparisons were conducted using the Bonferroni procedure.
